# The TRIM protein Mitsugumin 53 enhances survival and therapeutic efficacy of stem cells in murine traumatic brain injury

**DOI:** 10.1186/s13287-019-1433-4

**Published:** 2019-11-28

**Authors:** Fangxia Guan, Tuanjie Huang, Xinxin Wang, Qu Xing, Kristyn Gumpper, Peng Li, Jishi Song, Tao Tan, Greta Luyuan Yang, Xingxing Zang, Jiewen Zhang, Yuming Wang, Yunlei Yang, Yashi Liu, Yanting Zhang, Bo Yang, Jianjie Ma, Shanshan Ma

**Affiliations:** 1 School of Life Sciences, Zhengzhou University, Zhengzhou, 450001 Henan China; 2 The First Affiliated Hospital of Zhengzhou University, Zhengzhou, 450052 Henan China; 3 Henan Provincial People’s Hospital, No. 7 Weiwu Road, Zhengzhou, 450003 Henan China; 4 Department of Surgery, Davis Heart and Lung Research Institute, The Ohio State University, Columbus, OH 43210 USA; 5 Stuyvesant High School, 345 Chambers St, New York, NY 10282 USA; 6 Department of Microbiology and Immunology, Einstein College of Medicine, 1300 Morris Park Ave, Bronx, NY 10461 USA; 7 Department of Medicine and Neuroscience, Einstein College of Medicine, 1300 Morris Park Ave, Bronx, NY 10461 USA

**Keywords:** Neuroprotection, Mitsugumin 53, Stem cells, Traumatic brain injury, PI3K-Akt-GSK3β

## Abstract

**Background:**

Traumatic brain injury (TBI) is a common neurotrauma leading to brain dysfunction and death. Human umbilical cord-derived mesenchymal stem cells (hUC-MSCs) hold promise in the treatment of TBI. However, their efficacy is modest due to low survival and differentiation under the harsh microenvironment of the injured brain. MG53, a member of TRIM family protein, plays a vital role in cell and tissue damage repair. The present study aims to test whether MG53 preserves hUC-MSCs against oxidative stress and enhances stem cell survival and efficacy in TBI treatment.

**Methods:**

In this study, we performed a series of in vitro and in vivo experiments in hUC-MSCs and mice to define the function of MG53 enhancing survival, neurogenesis, and therapeutic efficacy of stem cells in murine traumatic brain injury.

**Results:**

We found that recombinant human MG53 (rhMG53) protein protected hUC-MSCs against H_2_O_2_-induced oxidative damage and stimulated hUC-MSC proliferation and migration. In a mouse model of contusion-induced TBI, intravenous administration of MG53 protein preserved the survival of transplanted hUC-MSCs, mitigated brain edema, reduced neurological deficits, and relieved anxiety and depressive-like behaviors. Co-treatment of MG53 and hUC-MSCs enhanced neurogenesis by reducing apoptosis and improving PI3K/Akt-GSK3β signaling.

**Conclusion:**

MG53 enhances the efficacy of hUC-MSCs in the recovery of TBI, indicating that such adjunctive therapy may provide a novel strategy to lessen damage and optimize recovery for brain injury.

## Introduction

Traumatic brain injury (TBI) is a common neural trauma that often initiates from an external force followed by a secondary neural injury, causing severe physical, psychological, and cognitive impairments [1, 2]. Several experimental and clinical studies have shown that stem cell transplantation can exert beneficial effects in TBI, acting via paracrine factors and providing cell replacement [3, 4]. Mesenchymal stem cells (MSCs) can be easily obtained from several tissues and characterized by self-renewal and differentiation potential. However, according to International Society for Cellular Therapy (ISCT) criteria, the isolated MSCs are heterogeneous, which contain stem cells, committed progenitors, and differentiated cells [5]. Though the nature of MSCs remains unclear, nonclonal stromal cultures currently serve as sources of putative MSCs, representing a promising therapy for neurodegenerative diseases. hUC-MSCs have advantages over embryonic stem cells, bone marrow, or adipose-derived mesenchymal stem cells in cell therapy for several reasons. hUC-MSCs are easily obtained and have fast proliferation rate and low immunogenicity, as well as low risk of teratoma formation [6, 7]. However, the migration and survival of transplanted stem cells are hindered by excessive reactive oxygen species (ROS) generated under conditions of tissue ischemia and inflammation [8, 9].

MG53, a member of the tripartite motif (TRIM) protein family, plays an essential role in cell membrane repair and tissue damage recovery [10–13]. Genetic ablation of MG53 results in defective membrane repair and tissue regeneration capacity [10, 11, 14, 15]. Recombinant human MG53 (rhMG53) protein could protect various cell types against membrane disruption and ameliorate the pathologies associated with muscular dystrophy [16], acute lung injury [17], myocardial infarction [18], and acute kidney injury [19] in animal models. Additionally, rhMG53 protects cultured neural cells from injury in vitro, and intravenous delivery of rhMG53 penetrates the blood-brain barrier to protect against ischemic brain injury [20]. In muscle membrane repair, MG53 binds to phosphatidylserine (PS) and interacts with caveolin-3 (Cav-3) to mediate vesicle accumulation at injury sites involved in patching the membrane [10, 11, 21]. In tissue repair, MG53 interacts with p85 as well as CaV3 and activates the pro-survival RISK pathway (including PI3K/Akt/GSK3β cascade and ERK1/2 pathway) to protect ischemic brain injury and myocardial damage [15, 20, 22]. However, it is unknown whether rhMG53 can enhance the viability and neural repair of hUC-MSCs in TBI and the mechanism remains unclear.

In this study, we tested whether rhMG53 can improve the survival of hUC-MSCs for the treatment of TBI. Our results showed that the combination of rhMG53 and hUC-MSCs had synergistic effects in augmenting the therapeutic benefits to mitigate brain edema and to improve the cognitive function of mice subjected to TBI. Moreover, the mechanism is through the activation of PI3K/Akt-GSK3β signaling.

## Materials and methods

### Isolation, cultivation, and identification of hUC-MSCs

hUC-MSCs were isolated as previously described [23]. This study was approved by the Ethics Committees of the Zhengzhou University. Briefly, human umbilical cords were obtained postpartum from full-term healthy infants delivered via normal vaginal delivery after informed consent according to institutional guidelines under an approved protocol. Cells were cultured in DMEM/F12 containing 10% FBS, 1% penicillin-streptomycin, and 10 ng/ml of basic fibroblast growth factor in a 37 °C incubator with 5% CO_2_. Flow cytometer (Becton-Dickinson, USA) was used to identify the immuno-phenotypic characterization of hUC-MSCs. Passages 3–5 of the hUC-MSCs were used for experiments.

### Preparation of recombinant human MG53 protein

Purification of rhMG53 protein has been described previously [16]. rhMG53 was lyophilized and stored at 4 °C. For intravenous injection of rhMG53, the protein was diluted at 2 mg/ml in 0.9% sterile saline, filtered through a 0.22-μm filter, and injected via the tail vein.

### Cell proliferation, apoptosis, and senescence-associated β-galactosidase (SA-β-gal) detection

hUC-MSCs were plated in 96-well plates at a density of 3 × 10^3^ cells/well and incubated with various concentrations of H_2_O_2_ within 60% confluence at 0, 8, 16, 24, 32, and 40 h after H_2_O_2_ incubation; CCK-8 assay was performed as previously described [24]. The effect of rhMG53 at different concentrations (15, 30, 60, 120 μg/ml) on hUC-MSC proliferation was also detected by CCK-8 assay. The experiments were divided into 4 groups: CON group, MG53 group (30 μg/ml rhMG53), H_2_O_2_ group (200 μM), and H_2_O_2_ + MG53 group (hUC-MSCs were co-treated with 30 μg/ml rhMG53 and 200 μM H_2_O_2_ stimulation for 16 h). Annexin V/propidium iodide (PI) staining was performed as previously described [25]. The senescence of hUC-MSCs was evaluated using a SA-β-gal kit (GenMed, USA) as described previously [24].

### Transwell assay

Cell migration was detected by a Transwell assay [25]. Briefly, hUC-MSCs treated with rhMG53 (30 μg/ml) and/or H_2_O_2_ (200 μM, 16 h) were collected and resuspended in DMEM/F12 media without serum, and re-plated in transwell with 8.0 μm holes at a density of 1 × 10^5^ cells/ml, 300 μl/well. Twenty-four hours later, the cells were fixed with 4% paraformaldehyde for 10 min and stained with crystal violet for 30 min. The cells in the upper chamber were removed, and the migrated cells were detected as purple in color and counted under a microscope (Eclipse TE2000-E; Nikon, Japan).

### Detection of biomarkers of oxidative stress (GSH, SOD, and MDA)

In in vitro studies, hUC-MSCs were collected and supernatants were harvested after cell lysed by ultrasonication. In in vivo studies, peripheral blood of mice was harvested and the serum was collected and stored at − 80 °C. The SOD activity and concentration of GSH and MDA in the cell supernatant or serum were measured according to the kit manufacturer’s instruction (Jian Cheng Biological Engineering Institute, China).

### Mouse model of TBI

All procedures involving animals were conducted under the National Guidelines for Care and Use of Laboratory Animals and the Animal Care Guidelines issued by Zhengzhou University. The C57BL/6 male mice (10–12 weeks, 23–25 g) were housed in cages and maintained at 24 °C with a normal 12 h/12 h light-dark schedule. The mice had free access to food and water. A weight-drop model of TBI was established as previously described [1]. A successful model was confirmed when the mice showed transient limb twitching and apnea after the injury and relieved themselves in a few seconds. The mice were kept on a heat mat to maintain a rectal temperature at 37 ± 0.5° during the operation and recovery.

### Experimental design

A total of 210 mice were randomly divided into 5 groups: Sham, TBI, MG53, MSC, and MG53 + MSC (42 mice in each group). rhMG53 (3 mg/kg) and/or hUC-MSCs (1 × 10^6^ cells) in 100 μl were injected intravenously via tail vein of TBI mice for 3 times at 6, 30, and 54 h after injury. The Sham and TBI group were treated with the same volume of 0.9% saline (100 μl). The timeline for animal experiments is showed in Fig. 3a.

### Western blotting

Mice were scarified 3 days after TBI (*n* = 6/group). The brains were removed, and the lesion areas were isolated for protein preparation. Western blotting was carried out as previously described [22]. Equal amounts of protein (150 μg) were separated by sodium dodecyl sulfate-polyacrylamide gel electrophoresis and transferred to polyvinylidene fluoride membranes (Bio-Rad). The primary antibodies were MG53 (1:1000), Bcl-2 and Bax (Cell Signaling Technology, 1:200), Akt1, GSK-3β, ERK1/2, p-Akt1, p-GSK-3β, p-ERK1/2 (Cell Signaling Technology, 1:1000), and β-actin (Sango, 1:2000). The intensities of the resulting protein bands were quantified with Image J software (NIH, Bethesda, MD, USA).

### Modified neurological severity score (mNSS)

mNSS was performed by observers blinded to the treatments at 0, 1, 3, 7, 14, 21, and 28 days after TBI as previously described [26]. The mNSS is a composite of motor, sensory, balance, and reflex tests which has been widely employed in TBI studies. This test is suitable to evaluate long-term neurological outcome after unilateral brain injury [3]. The test was performed double blindly by examiners.

### Brain water content (edema)

The extent of brain edema after TBI was measured 3 days after TBI as described previously [27]. Briefly, whole brain specimens were harvested. Ipsilateral hemisphere and contralateral hemisphere were weighed immediately on an analytical balance to obtain the wet weight and dried for 24 h at 100 °C to obtain the dry weight. The formula for water content is as follows: percentage of water = (wet weight − dry weight)/wet weight.

### Morris water maze (MWM)

MWM was used to evaluate spatial learning and memory at day 28 after TBI as previously described [28, 29]. Before experimentation, mice were trained four times in 20-min intervals with the start position randomly changed. The mice were gently placed in the apparatus and allowed for 60 s to find the platform and stay on it for 10 s. If the mice could not find the platform in 60 s, they were gently guided to the platform and stayed on the platform for 10 s, and the latency time was recorded as 60 s. On the 28th day, the platform was removed, and the mice were placed in the opposite quadrant to the one previously described. The mice were towel dried and placed into a heated cage after each trial. Results were expressed as swimming tracks, times to cross the platform, latencies to platform, and times spent in the goal quadrant.

### Novel object recognition (NOR) test

Twenty-eight days post-TBI, we used an established protocol to assess each mouse with the NOR test [30–32]. The experiments were carried out in 27 days after TBI, and the mouse was placed in the box and habituated for 10 min. On the next day, two identical novel objects (black cubes, 4 × 4 × 4 cm^3^) were placed in the arena, and the mouse was allowed to explore the area for 10 min. After 1 h, one novel object (blue ball, 4 cm in diameter) and one old object (black cube) were placed in the box, and the mouse was allowed to explore for 5 min while being recorded by camera. We compared the total time spent exploring the old and new objects. The exploration time included time in direct contact with the objects and time within the object area; a discrimination index (total time spent with new object/total time devoted to exploration of objects) was also calculated for each mouse.

### Forced swim test (FST)

FST was used to assess depression of the mice. On day 28 post-TBI, the mice underwent the FST as previously described [32]. Mice were placed individually in clear cylindrical tanks (height = 25 cm; diameter = 22 cm) with 15 cm of water at 23 ± 1 °C. The trial was conducted for 6 min, and the period of immobility during the last 4 min was measured. The immobility time was defined as the animal floating on the surface of the water, only making minimal movements necessary to remain afloat, typically slow paddling of one foot.

### Tail suspension test (TST)

The TST was performed with each mouse 28 days post-TBI. Briefly, mice were suspended by the tail with a piece of adhesive tape (1 cm from the tip, 17 cm long) 55 cm above the desk. A camera corded the movement of the mouse for 3 min. Mice were considered immobile when they hung passively and motionless. The immobility time was calculated by subtracting the total amount of mobility time from the 180 s of test time. The method was according to a protocol described previously [33].

### Sucrose preference test (SPT)

The sucrose preference test was carried out at 28 days after TBI. The test was performed as described previously with some slight modifications [32]. Before the test, mice were put to the cage having two bottles of 50 ml sucrose solution (1%, w/v) for 3 days; then, one bottle of sucrose was replaced with water. After 4 days, the respective weights of the sucrose solution and water consumed were recorded, and the percent sucrose preference was calculated by using the following formula: Sucrose preference = [sucrose consumption in g/(water + sucrose consumption in g)] × 100%. Diminished preference for the sweet drink indicates depression-like behavior.

### Novelty suppressed feeding test (NSF)

At 28 days after TBI, NSF test was performed during a 5-min period [34]. The mice were weighed, and all food was removed from their cages. Water continued to be provided. Approximately 24 h after the removal of the food, the mice were transferred to the testing room, placed in a clean holding cage, and allowed to habituate for 30 min. The testing apparatus consisted of a Plexiglas box (45 × 45 × 20 cm) under an illuminated (approximately 1000 lx), soundproofed condition. The floor of the box was covered with 1 cm of wooden bedding. A small piece of mouse chow was placed in the center of the box on a white circular filter paper (11 cm in diameter). Each subject was placed in the corner of the testing arena, and the time until the first feeding episode was recorded. At the end of this period, the time to enter the center was counted.

### Open-field test (OFT)

The open-field test was conducted at 28 days after TBI with apparatus that consisted in a 45 cm × 45 cm wooden square surrounded by a 20-cm high wall. The bottom of the black box was divided into 25 squares (7 cm × 7 cm) with 2 mm white lines. During the test, the mice were placed in one of the four corners of the box and facing the wall. The mice were recorded for the total distance traveled and number of rearings (vertical standing of mice on two hind legs) within 3 min [35].

### In vivo PI (propidium iodide) staining

Three days after TBI, we diluted PI (10 mg/ml; Sigma-Aldrich, St Louis, MO, USA) in 0.9% NaCl and administered to mice by intraperitoneal injection at 0.4 mg/kg before sacrifice [29] to detect cell death in damaged areas of cerebral cortex of TBI mice. The sections were also stained with 4′, 6-diamidino-2-phenylindole (DAPI) to show total nuclei. Samples were observed and photographed under a fluorescence microscope (Eclipse TE2000-E; Nikon, Japan) at excitation and emission wavelengths of 535 nm and 617 nm, respectively.

### Tissue processing and immunofluorescence staining

At 3, 7, 14, and 28 days after TBI, mice were deeply anesthetized with isoflurane and perfused with phosphate-buffered saline (PBS) followed by 4% paraformaldehyde via cardiac puncture. All solutions were maintained at pH 7.4 and 4 °C. Brains were removed and stored in 4% paraformaldehyde overnight and then transferred to 30% sucrose in PBS for another 48 h. Frozen serial coronal brain sections were sliced at 10 μm on a cryostat (Leica, Germany).

Mouse anti-human nuclei monoclonal antibody (MAB1281) was applied for immunostaining to trace the migration of hUC-MSCs in the brain of TBI mice by 3 days post-TBI. Immunofluorescence staining for glial fibrillary acidic protein (GFAP), doublecortin (DCX), and neuronal nuclei (NeuN) was used to detect the degree of hippocampal neurogenesis in TBI mice as described previously [36]. After being blocked with 10% goat serum for 45 min, brain sections (*n* = 6 mice/group) were incubated with MAB1281 (1:50, Merck Millipore), GFAP (1:50, Cell Signaling Technology), DCX (1:50, Santa), and NeuN (1:50, Abcam) at 4 °C overnight and then with FITC/CY3-conjugated secondary antibody (1:1000, Sangon Biotech) for 1 h at room temperature. Stained sections were examined under a microscope (Eclipse TE2000-E; Nikon). The quantification of MAB1281-, GFAP-, DCX-, or NeuN-positive cells was analyzed using Image J software (NIH, Bethesda, MD, USA) and divided by the total area in the image field (mm^2^).

### Cresyl violet staining and lesion volume

On day 28 after TBI, coronal brain sections were stained with Cresyl violet to detect Nissl bodies as described previously [37]. Briefly, the brains were continuously coronally sectioned from the lesion with the edge of 2.0 mm. Each slice was 20 μm thick and randomly selected from 10 consecutive slices. Using the Image J software (NIH, Bethesda, MD, USA), the brain damage volume was calculated: brain damage volume (mm^3^) = average lesion area × number of brain slices (10) × 0.02 [37].

### Polymerase chain reaction (PCR) and quantitative real-time PCR (qRT-PCR)

To confirm that hUC-MSCs could migrate to brain after intravenous injection, the presence of human-specific DNA (a 479-bp fragment of a highly repetitive α-satellite DNA sequence of the centromere region of human chromosome 17) in the hippocampus of TBI mice was detected 3 days after administration by using PCR technique. Genomic DNA was extracted from brain tissues using a DNA tissue Kit (Qiagen, USA) and quantified by a Nanodrop 2000 spectrophotometer (Thermo Fisher, USA). After PCR, the products were detected by 1% agarose gel electrophoresis, stained with ethidium bromide (Sigma, USA), and visualized using UV trans-illumination. Primers for human-specific PCR were as follows: forward-5′-GGGATAATTTCAGCTGACTAAACAG-3′, reverse-5′-AAACGTCCACTTGCAGTTCTAG-3′; GAPDH: forward-5′-GGTGAAGGTCGGTGTGAAC-3′, reverse-5′-CTCTGACCTGTGCCGTTGAA-3′. The program consisted of 40 cycles of denaturation for 30 s at 95 °C, annealing for 30 s at 58 °C, and extension for 40 s at 72 °C [38].

For qRT-PCR, brains were collected at 28 days after TBI, and total RNA were isolated to check the transcription level of NGF and BDNF [39]. The primer sequences were as follows: NGF—forward-5′-TACAGGCAGAACCGTACACAGATAG-3′, reverse-5′-CAGTGGGCTTCAGGGACAGA-3′; BDNF—forward-5′-CATAGACAAAAGGCACTGGAACTC-3′, reverse-5′-TAAGGGCCCCGAACATACGAT-3′. The 2^-ΔΔCT^ method was used to obtain relative fold change of target gene expression normalized by the housekeeping gene GAPDH.

### Statistical analysis

Data are presented as mean ± SEM. Two-way ANOVA was used to analyze all behavioral tests between and among the treatment groups. In anatomical and biochemical studies, one-way or two-way ANOVA was used to compare multiple groups. A Bonferroni post hoc analysis was used to determine whether differences were significant. Differences between two groups were tested with the two-tailed Student’s t-test. The criteria for statistical significance were *p* < 0.05.

## Results

### rhMG53 protects cultured hUC-MSCs against oxidative damage and promotes cell migration

**Fig. 1:**
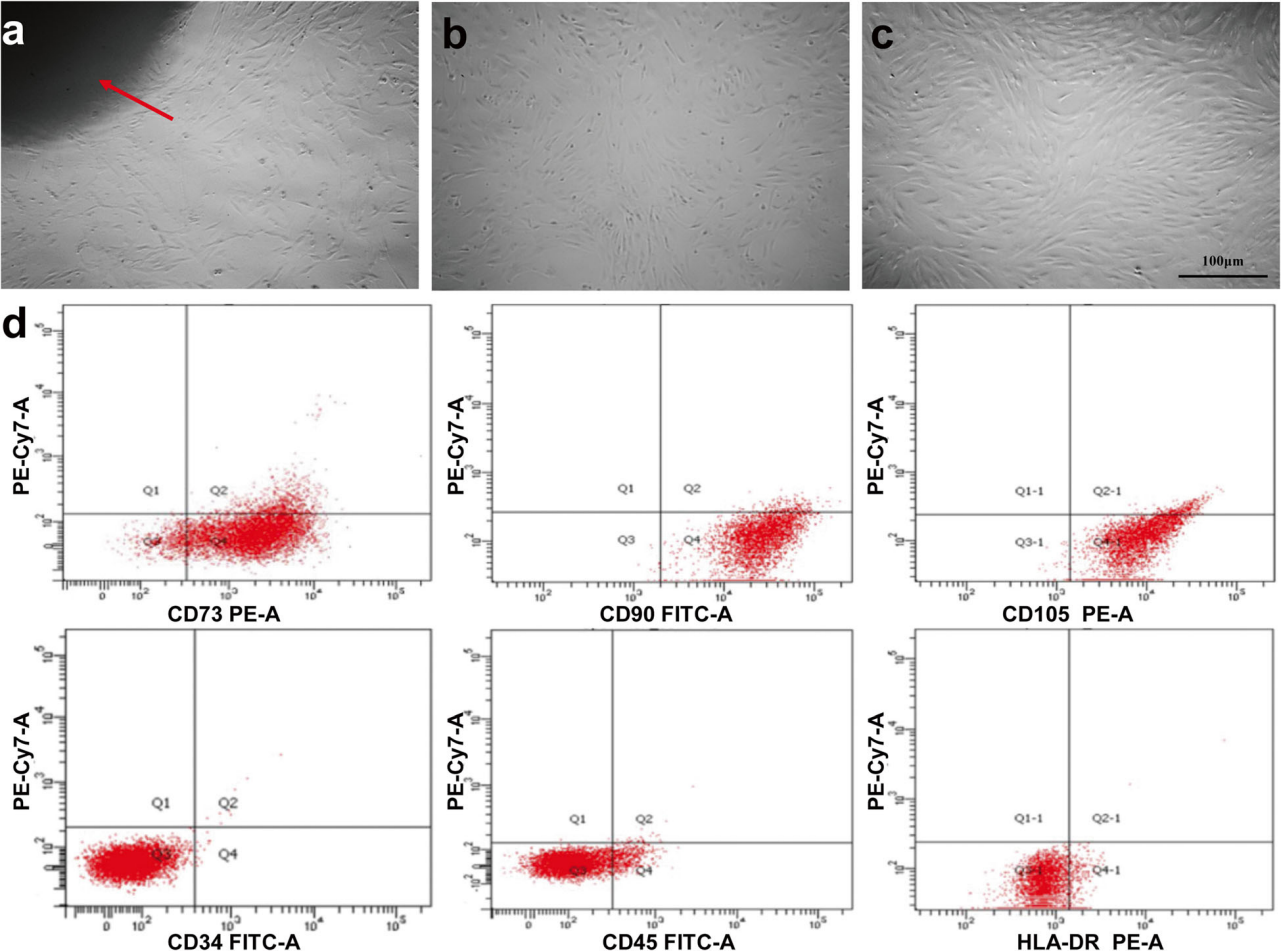
Growth morphology and immuno-phenotypic characterization of hUC-MSCs. **a** Wharton’s jelly tissue pieces (red arrow) were plated and cultured for 2 weeks, in order to grow out primary hUC-MSCs (P0) from the tissues. **b** After 2 days in culture, passage-3 (P3) hUC-MSCs displayed a spindle-shaped or fibroblast-like morphology. **c** After 4 days in culture, morphology of P3 hUC-MSCs changed to a homogeneous fibroblastoid cell type with a smooth border. Scale bar = 100 μm. **d** Flow cytometric analysis were used to characterize the P3 hUC-MSCs. hUC-MSCs were positive for CD73, CD90, and CD105 and negative for hematopoietic lineage markers CD34, CD45, and HLA-DR

hUC-MSCs were derived from Wharton’s jelly and displayed fibroblast-like morphology approximately 2 weeks after primary culture (Fig. 1a–c). Flow cytometric analysis showed that the isolated hUC-MSCs were positive for CD73, CD90, and CD105 and very low or negative for hematopoietic lineage markers CD34, CD45, and HLA-DR (Fig. 1d).

**Fig. 2:**
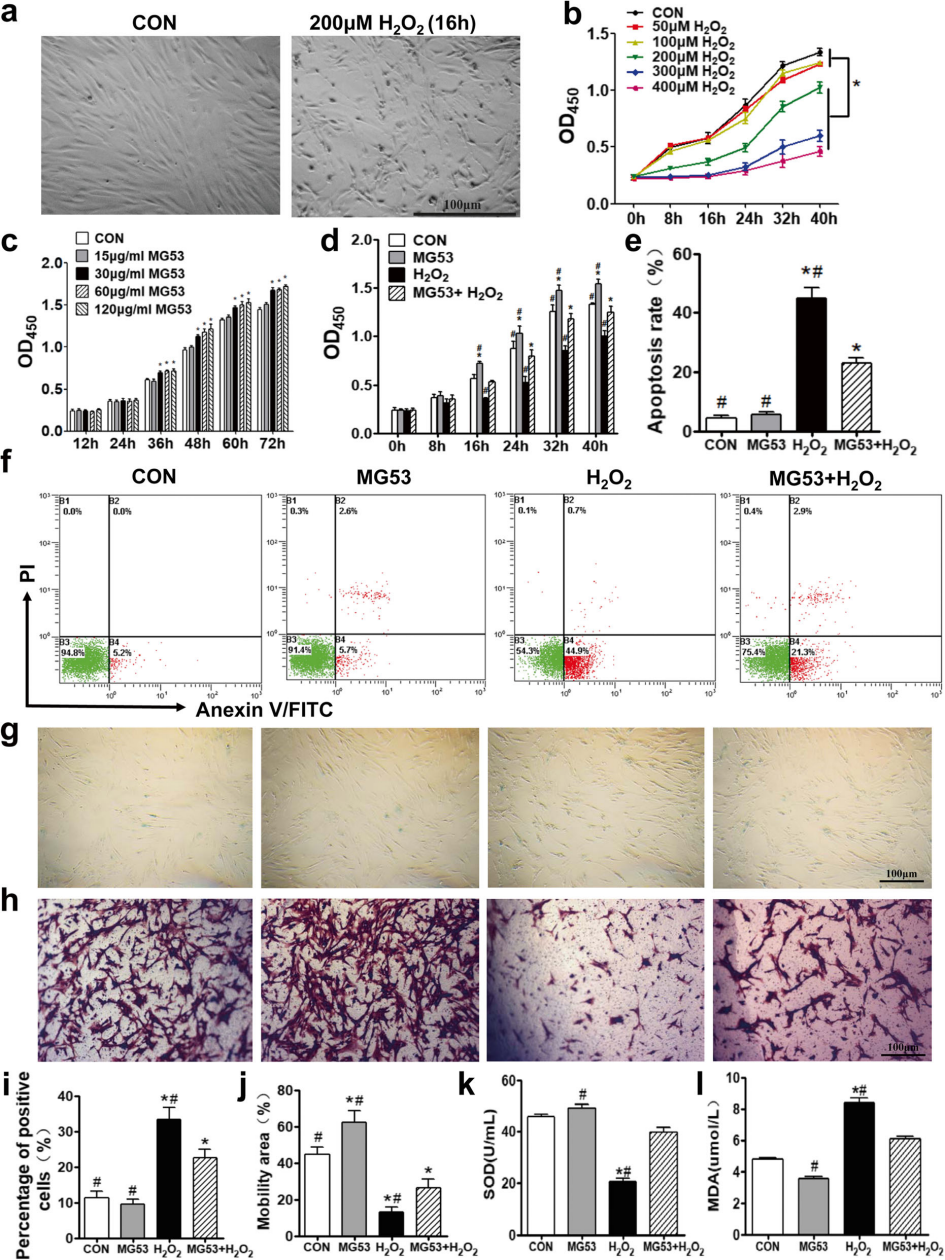
rhMG53 lessens H_2_O_2_-induced oxidative injury to hUC-MSCs and promotes cell migration. **a** Representative images of hUC-MSCs with and without 200 μM H_2_O_2_ treatment. **b** Time- and dose-dependent effects of H_2_O_2_ on hUC-MSCs. Cells were cultured in 0, 50, 100, 200, 300, or 400 μM H_2_O_2_, and OD450 was measured at 0, 8, 16, 24, 32, and 40 h post-treatment. Two hundred micromolar H_2_O_2_ was used for subsequent experiments to induce hUC-MSC oxidative damage. **c** Dose-dependent effects of MG53 on hUC-MSCs. Thirty micrograms per milliliter of rhMG53 was chosen for our in vitro experiments. **d** rhMG53 facilitates hUC-MSC proliferation and protects against H_2_O_2_-induced injury. **e** Quantification of apoptosis rate from Annexin V-FITC/PI flow cytometry. **f** Apoptosis of hUC-MSCs was detected and analyzed by Annexin V-FITC and PI double staining and flow cytometry as well. **g** Cell senescence was evaluated using a SA-β-gal kit. Senescent cells were dyed blue. **h** Transwell assay was used to assess cell migration. Migrated cells were stained with CV. Scale bar = 100 μm. Quantification of cell senescence (**i**) and migration (**j**). SOD activity (**k**) and MDA content (**l**) were measured from hUC-MSC lysates. Data are presented as mean ± SEM. *n* = 6 per group. *p < 0.05, compared with CON group; ^#^*p* < 0.05, compared with MG53 + H_2_O_2_ group

In order to determine how sensitive hUC-MSCs were to oxidative stress, hUC-MSCs at third passage (P3) were treated with varying concentrations of H_2_O_2_. Results showed that H_2_O_2_ exhibited dose- and time-dependent inhibitory effects on the viability of hUC-MSCs at a concentration range of 0–400 μM (Fig. 2a, b). Based on the data, after treatment for 16 h, the half maximal inhibitory concentration of hUC-MSCs was 200 μM H_2_O_2_. So, 200 μM H_2_O_2_ was selected for the subsequent experiments to induce oxidative damage to hUC-MSCs.

In addition, the proliferation of hUC-MSCs was significantly improved as the concentration of rhMG53 increased (≥ 30 μg/ml) (*p* < 0.05, Fig. 2c). Considering the dose effect of rhMG53 and previous studies, 30 μg/ml rhMG53 was chosen for our further in vitro experiments [16]. The protective effects of rhMG53 on H_2_O_2_-induced oxidative damage to hUC-MSCs were then examined. CCK8 results showed that rhMG53 promoted proliferation of hUC-MSCs and also mitigated the inhibitory effects of H_2_O_2_ (*p* < 0.05, Fig. 2d). H_2_O_2_-induced cell apoptosis and senescence of hUC-MSCs were significantly recovered by rhMG53 (*p* < 0.05, Fig. 2e, f and Fig. 2g, i), while the reduction of cell migration triggered by H_2_O_2_ was partially improved by rhMG53 (*p* < 0.05, Fig. 2h, j).

Finally, superoxide dismutase (SOD) activity and malonyldialdehyde (MDA) content were measured to quantify the effects of H_2_O_2_ and rhMG53 treatments on oxidative damage to hUC-MSCs. Upon treatment with H_2_O_2_, SOD activity was significantly decreased (*p* < 0.05, Fig. 2k), and MDA content was significantly increased (*p* < 0.05, Fig. 2l), while rhMG53 could significantly reverse both effects (*p* < 0.05, Fig. 2k, l). These results showed that rhMG53 can protect cultured hUC-MSCs against oxidative damage and promote cell migration by decreasing oxidative stress induced by H_2_O_2_ in vitro.

### rhMG53 enables hUC-MSC survival and entry into mouse brains

**Fig. 3:**
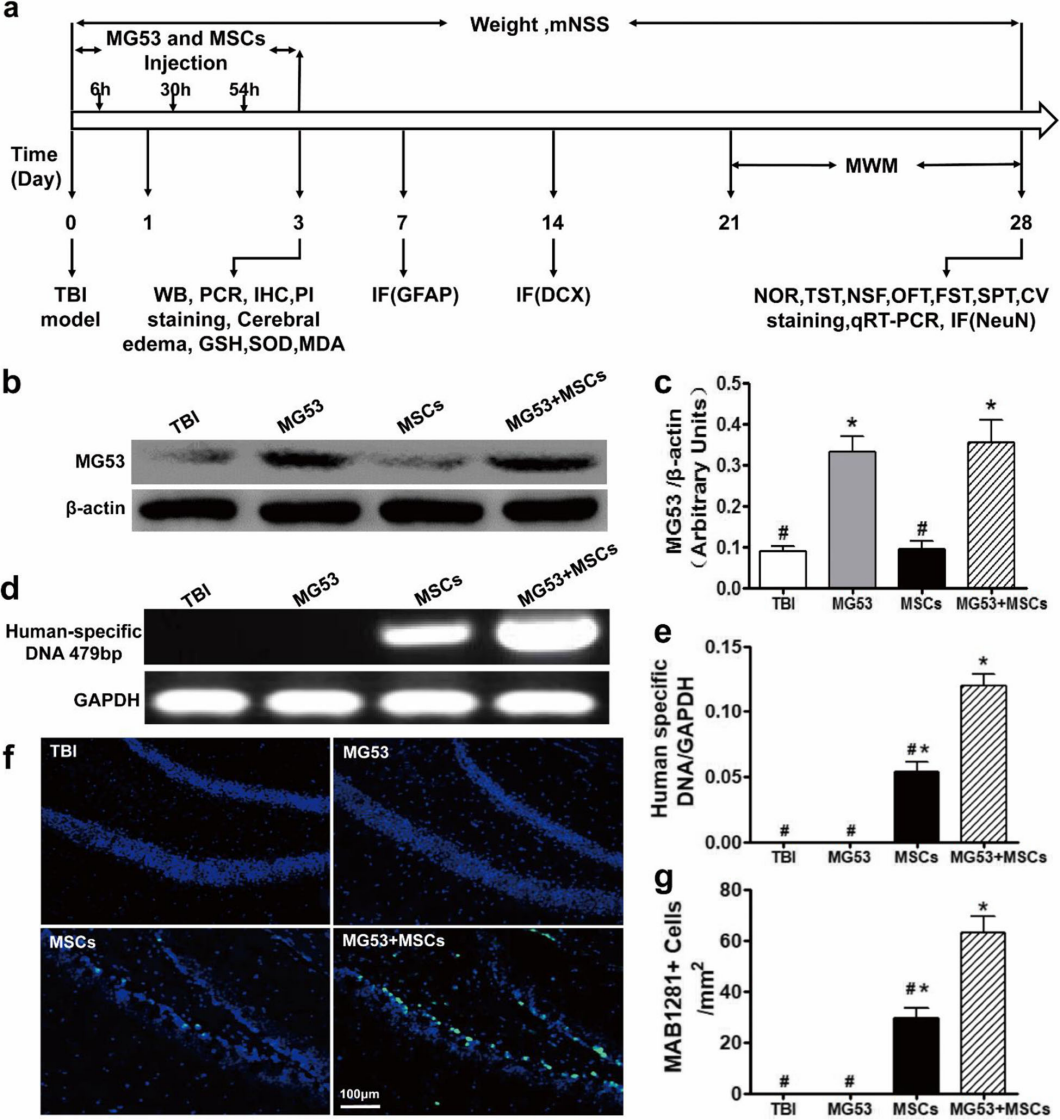
rhMG53 crosses the blood-brain barrier and enhances the migration of hUC-MSCs in the brains of TBI mice. **a** The timeline for animal experiments. **b** Western blot of MG53 in mouse brains following TBI. **c** Densitometric analysis of MG53 protein levels from multiple mice. **d** PCR results of the human-specific DNA (479 bp) in the hippocampus of the TBI mice. **e** Quantification of the PCR results for human-specific DNA in mouse hippocampus. **f** Representative images of immunohistochemical staining with MAB1281 to identify the content of hUC-MSCs in the hippocampus. Scale bar = 100 μm. **g** Quantification of MAB1281+ cells in the hippocampus. Data are presented as mean ± SEM. *n* = 6 per group. *p < 0.05, compared with TBI group; ^#^*p* < 0.05, compared with MG53 + MSC group

Before assessing the protective effects of rhMG53 and hUC-MSCs on TBI, whether rhMG53 could aid the homing of hUC-MSCs in the mouse brain was examined. The experimental design and timeline for animal experiments are shown in Fig. 3a. As shown in Fig. 3b and quantified in Fig. 3c, TBI induced the appearance of MG53 in the brain tissue, which was not detected in the brain under physiological conditions [20]. The expression of endogenous MG53 in the injured brain tissue likely reflected an increased permeability of blood-brain barrier (BBB). Further elevation of rhMG53 was observed after intravenous administration of the exogenous protein to mice subjected to TBI (Fig. 3b), indicating that rhMG53 can cross the BBB to target the injured brain tissue, which is similar to our previous observation in a mouse model of ischemic brain injury [20].

The presence of human-specific DNA in the mouse brain tissues was used as a marker to determine the homing of hUC-MSCs by PCR analysis [4, 38]. As expected, no PCR product of human-specific DNA was detected in brain lesions derived from mice subjected to TBI, with or without rhMG53 treatment. In TBI mice receiving hUC-MSCs, abundant human-specific PCR product was detected (Fig. 3d). Moreover, TBI mice receiving co-treatment of rhMG53 + hUC-MSCs displayed significantly more human DNA in their brain lesions (*p* < 0.05, Fig. 3d, e).

Immunohistochemical staining was subsequently used to investigate the presence of hUC-MSCs in the mouse hippocampus. MAB1281 is an anti-human nuclear monoclonal antibody, which specifically reacts with the nucleus of all human cells [3]. MAB1281-positive cells were observed in the brain lesions derived from mice that received either hUC-MSCs or rhMG53 + hUC-MSCs within 3 days after hUC-MSC transplantation, but not in TBI mice receiving saline as control or rhMG53 alone (Fig. 3f). Quantitative analysis showed that the number of MAB1281-positive cells in rhMG53 + hUC-MSC group (63.5 ± 12.0 cells) was significantly higher than that in hUC-MSC group (31 ± 9.5 cells, *p* < 0.05, Fig. 3g). These results indicated that rhMG53 treatment either preserved the survival of hUC-MSCs in mouse brain following TBI or enhanced their ability to migrate into the brain by crossing the BBB.

### rhMG53 and hUC-MSCs mitigate brain edema and improve survival signaling of brain tissues in mouse model of TBI

**Fig. 4:**
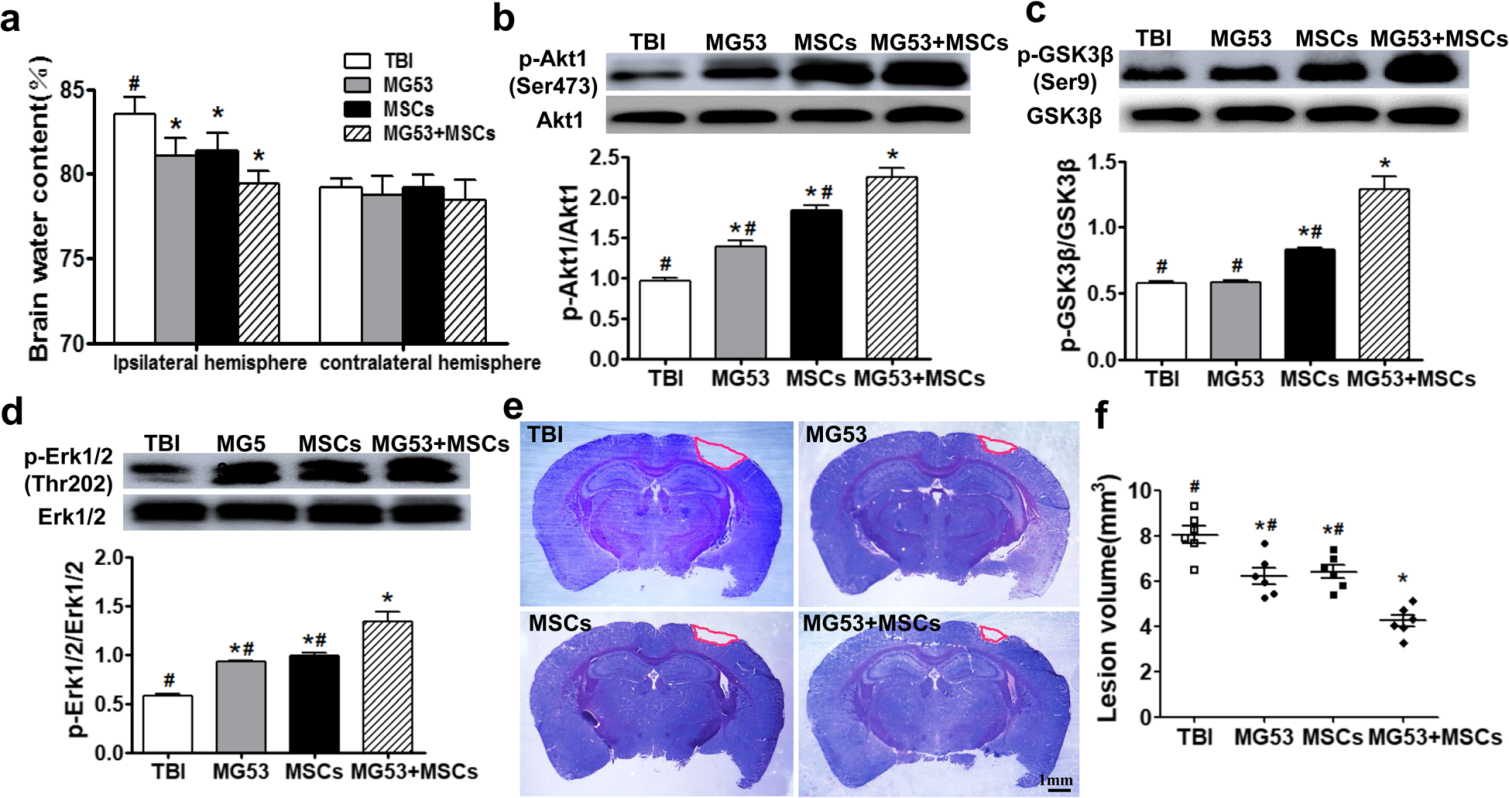
rhMG53 and hUC-MSCs decrease brain edema and reduce brain lesion volume after TBI by activating PI3K/Akt-GSK3β and ERK signaling. **a** Percentage of brain water content between ipsilateral hemisphere and contralateral hemisphere at day 3 post-TBI. Representative immunoblots and statistical analysis for phosphorylated and total Akt1 (**b**), GSK3β (**c**), and ERK 1/2 (**d**) in lysates from the injured brain tissues 3 days after TBI. **e** Cresyl violet staining of brain sections at 28 days post-TBI. Injured areas lack staining and are circled in red. Scale bar = 1 mm. **f** Quantification of lesion volume from the Cresyl violet-stained brains. Data for all graphs were presented as mean ± SEM. *n* = 6 per group. *p < 0.05, compared with TBI group; ^#^*p* < 0.05, compared with MG53 + MSC group

At 3 days post-TBI, brain edema of the ipsilateral hemisphere was significantly alleviated in the MG53, MSC, or MG53 + MSC group than that in TBI group (*p* < 0.05, Fig. 4a). However, none of the treatments had a significant impact on the degree of edema in the contralateral hemisphere when compared to TBI group (*p* > 0.05, Fig. 4a).

Fresh brain tissues derived from the injured lesions at 3 days after TBI were used for Western blotting analysis. As shown in Fig. 4b–d, improved cell survival signaling of p-Akt1 (Ser473), p-GSK3β (Ser9), and p-ERK1/2 (Thr202) was observed in MG53, MSC, or MG53 + MSC group (*p* < 0.05), whereas the total expressions of Akt1, GSK3β, and ERK1/2 were not affected (Fig. 4b–d, *p* > 0.05).

Then, the effects of the rhMG53 and hUC-MSC treatments on long-term brain structure were evaluated using Cresyl violet staining (Fig. 4e). At 28 days after TBI, results showed that the size of the lesion in the ipsilateral hemisphere was significantly reduced than those in the MG53 and MSC group. Moreover, co-treatment with rhMG53 and hUC-MSCs led to further reduction of the brain lesions (*p* < 0.05, Fig. 4f). Together, these results indicated that co-administration of rhMG53 and hUC-MSCs resulted in both short-term and long-term improvements of brain structure in the mouse model of TBI.

### rhMG53 and hUC-MSCs alleviate oxidative stress, decrease neural apoptosis, and promote neurogenesis after TBI

**Fig. 5:**
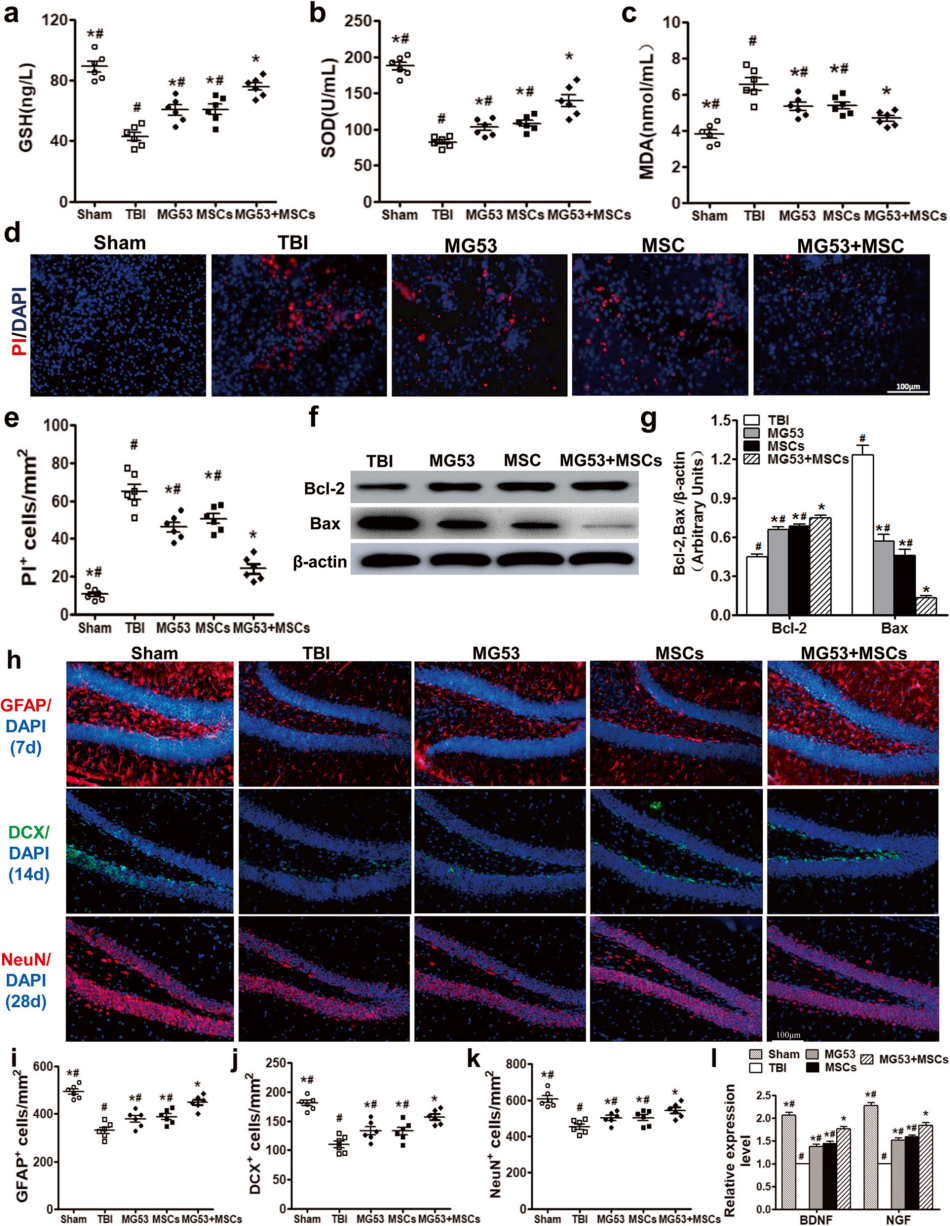
rhMG53 and hUC-MSCs reduce oxidative stress and cell death and increase neurogenesis after TBI. Quantification of the concentration of GSH (**a**) and SOD (**b**) and activity of MDA (**c**) at day 3 post-TBI. **d** PI staining in the cerebral cortex of TBI mice as a marker for cell death at 3 days post-TBI. Scale bar = 100 μm. **e** Quantification of the number of PI-positive cells in the four groups. Western blotting (**f**) and densitometric analysis (**g**) of Bcl-2 and Bax in the hippocampus of different TBI mice. **h** Immunofluorescence staining of GFAP^+^, DCX^+^, and NeuN^+^ cells in the brain of the mice. Scale bar = 100 μm. Quantification of the number of GFAP^+^ (**i**), DCX^+^ (**j**), and NeuN^+^ (**k**) cells in the four groups. **l** qRT-PCR for BDNF and NGF. Data were presented as mean ± SEM. *n* = 6 per group. *p < 0.05, compared with TBI group; ^#^*p* < 0.05, compared with MG53 + MSC group

Often, TBI and subsequent brain edema can lead to an increase in oxidative stress in the surrounding tissue, resulting in exacerbated brain damage [40]. To examine the potential anti-oxidative effects of rhMG53 and hUC-MSCs in the TBI mice brain, the concentrations and activities of oxidative markers (GSH, SOD, and MDA) in the hippocampus at 3 days after injury were quantified. rhMG53 or hUC-MSCs caused significant elevation of GSH and SOD (*p* < 0.05, Fig. 5a, b) and a decrease of MDA content (*p* < 0.05, Fig. 5c). But, the changes of GSH, SOD, and MDA in MG53 + MSC group were most significant (*p* < 0.05, Fig. 5a–c). These data suggest that co-treatment of rhMG53 and hUC-MSCs reduced the degree of oxidative stress in the hippocampus of mice with TBI.

PI staining was used to quantify the number of dying cells in the injured brain tissue at 3 days after TBI (Fig. 5d). The number of PI-positive cells in MG53 or MSC groups was significantly reduced than that of TBI mice. The most notable decrease of PI-positive cells occurred in MG53 + MSC group (*p* < 0.05, Fig. 5e), indicating that rhMG53 and hUC-MSCs ameliorated neuronal cell death. This result was further confirmed by Western blotting, which showed that the anti-apoptosis associated protein (Bcl-2) was increased while pro-apoptosis protein (Bax) was decreased in the MG53, MSC, and MG53 + MSC group (*p* < 0.05, Fig. 5f, g).

Furthermore, immunofluorescence staining was used to evaluate the potential benefits of rhMG53 and hUC-MSC treatment on neurogenesis following brain injuries. As shown in Fig. 5h–k, there were more GFAP^+^ cells at day 7, DCX^+^ neurons at day 14, and NeuN^+^ neurons at day 28 in MG53, MSC, and MG53 + MSC groups than those in TBI group (*p* < 0.05), but less than that in Sham group. qRT-PCR at 28 days post-TBI showed upregulated expressions of brain-derived neurotrophic factor (BDNF) and nerve growth factor (NGF) in all treated groups and most obviously in the MG53 + MSC group (*p* < 0.05, Fig. 5l). These data suggest that rhMG53 and hUC-MCSs not only reduced oxidative stress and subsequent cell death in injured brain tissue but also promoted neurogenesis.

### rhMG53 and hUC-MSC treatment improve neurologic function of mice after TBI

**Fig. 6:**
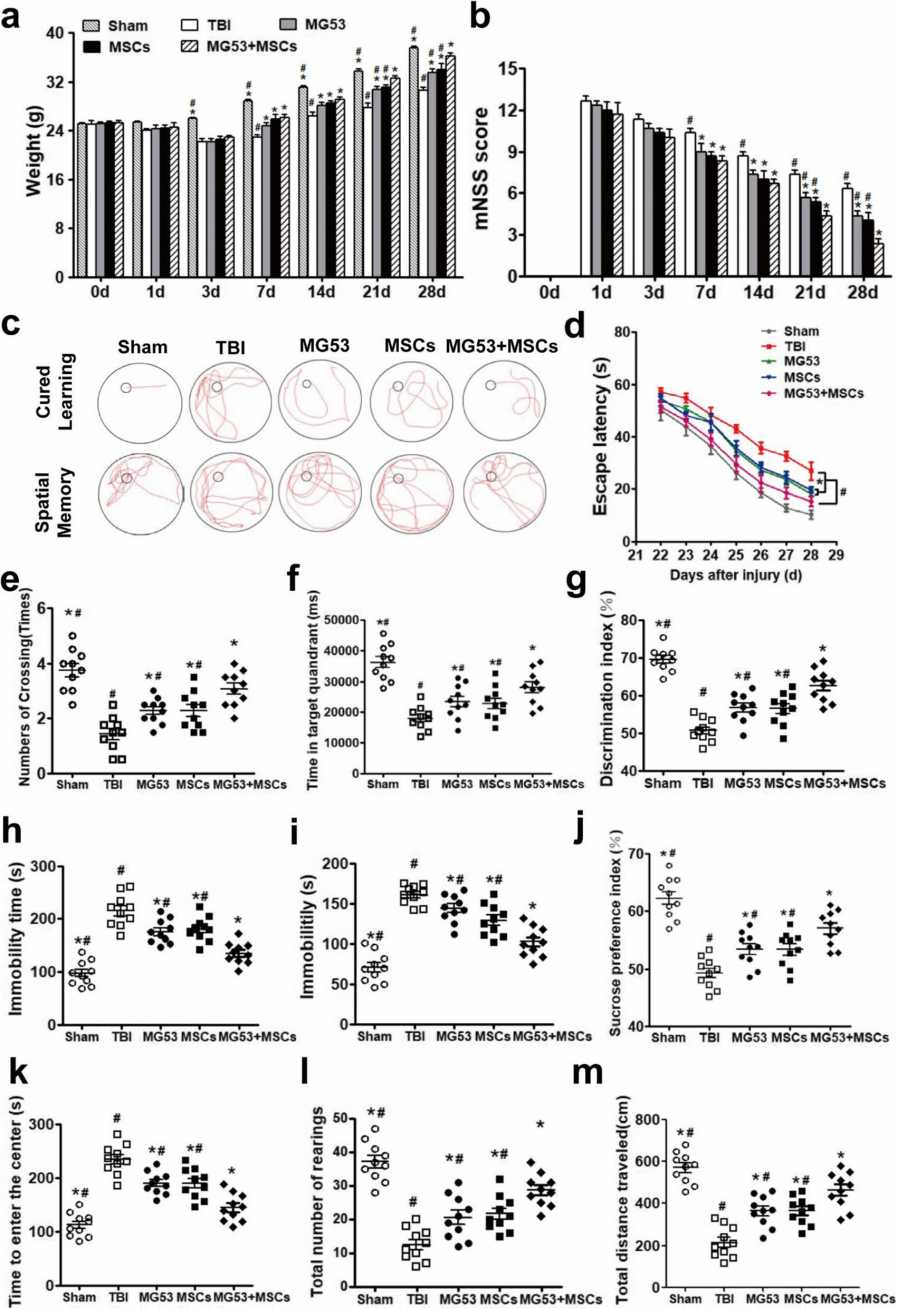
rhMG53 and hUC-MSC treatment reverse neurologic function impairments and relieve anxiety and depressive-like behavior in mice after TBI. **a** Time-dependent changes in body weight of TBI mice subjected to different treatments. **b** mNSS assessments at different stages of TBI recovery. **c** Representative swimming trajectories during the MWM test. Quantification from the MWM was for escape latency (**d**), number of crossings (**e**), and time spent in the target quadrant (**f**). **g** The discrimination index in the novel object recognition test. Immobility time was assessed using forced swimming test (**h**) and the tail suspension test (**i**). The sucrose preference index (**j**) was measured using sucrose preference test. **k** Assessment of conflict-based anxiety as time to enter the center during novelty suppressed feeding test. Assessment of exploratory drive. Total number of rearings (**l**) and total distance traveled (**m**) were recorded during open-field test. Data were presented as mean ± SEM. *n* = 10 per group. *p < 0.05, compared with TBI group; ^#^*p* < 0.05, compared with MG53 + MSC group

Body weight was increased more in the MG53 + MSC group, but least in the TBI group compared with the other groups from day 14 to day 28 after TBI (*p* < 0.05, Fig. 6a). Compared with TBI group, mice in MSC, MG53, and MG53 + MSC group exhibited significantly lower mNSS scores from day 7 to day 28 after TBI, and lowest in MG53 + MSC group at day 21 to 28 (*p* < 0.05, Fig. 6b). These results indicated treatment with rhMG53 and hUC-MSCs improved resilience and recovery after TBI.

At day 28 post-TBI, the Morris water maze (MWM) was used to examine the spatial learning and cognitive function of the mice (Fig. 6c). Mice in MG53 and/or MSC groups performed significantly better than TBI group, as indicated by shorter latency to reach the platform (Fig. 6d), more crossing numbers (Fig. 6e), and more time spent in the target quadrant (*p* < 0.05, Fig. 6f). In addition, compared with TBI mice, MG53 + MSC mice demonstrated a higher discrimination index in the NOR test, with significantly improved cognition in MG53 + MSC group (*p* < 0.05, Fig. 6g).

### rhMG53 and hUC-MSCs relieve anxiety and depressive-like behavior in TBI mice

Anxiety and depression were also assessed in the TBI mice at day 28 using forced swim test (FST), tail suspension test (TST), sucrose preference test (SPT), novelty suppressed feeding test (NSF), and open-field test (OFT). When subjected to rhMG53 and/or hUC-MSCs, TBI mice exhibited a significant decrease in immobility time in FST and the TST (*p* < 0.05, Fig. 6h, i), both of which are considered signs of depressive behavior [32]. SPT is another common method of assessing depression through anhedonia, the inability to feel pleasure [40]. Compared with TBI group, rhMG53 and/or hUC-MSC treatment significantly increased the sucrose preference index, indicating less anhedonia and vulnerability or resistance to stress (*p* < 0.05, Fig. 6j).

In addition, rhMG53- or hUC-MSC-treated mice exhibited decreased anxiety as demonstrated by a decrease in the time to enter the disk containing food, whereas that of co-treatment of rhMG53 and hUC-MSCs was least at 28 days after TBI (*p* < 0.05, Fig. 6k). Similarly, the total number of rearings and the total distance traveled in MG53 or MSC group were greatly increased in the OFT, and the most effective improvement was detected in MG53 + MSC group (*p* < 0.05, Fig. 6l, m). Taken together, our results indicated that rhMG53 and hUC-MSC treatment relieved anxiety and depressive-like behavior after TBI and the combination treatment resulted in the significant beneficial effects.

## Discussion

According to the World Health Organization, TBI is a major health problem worldwide. Accumulating studies prove evidence that stem cell transplantation is a promising approach for TBI treatment [41]. It has been shown that intravenously administered MSCs can migrate to the brain lesion to improve neural function in TBI [42, 43]. However, the therapeutic efficacy of MSCs in TBI remains unsatisfactory due to limited migration and survival of transplanted stem cells in brain. rhMG53, a protein critical for membrane repair, was found to enhance the survival of hUC-MSCs after ox-LDL injury [44], as well as facilitate the repair of ischemia-reperfusion injury to the brain [20]. Our results presented evidence that rhMG53 could preserve survival of hUC-MSCs in vitro and in vivo and enhance therapeutic efficacy for TBI treatment.

Considering that rhMG53 has a short half-lifetime of ~ 1.5 h in the blood circulation after intravenous or subcutaneous administration [16, 19], repetitive administrations of rhMG53 are required. In our studies, rhMG53 and/or hUC-MSCs were respectively injected three times through intravenous administration after TBI.

TBI primarily leads to edema of the brain, which contributes to a range of complications and secondary injuries [1, 45, 46]. Our previous studies showed that mice treated by Wharton’s jelly tissue (which is able to produce hUC-MSCs) gained body weight than vehicle group during the 28 days after TBI [27]. We found that rhMG53 and hUC-MSCs could mitigate brain edema, and when applied together, edema was further alleviated in the TBI mice. Furthermore, rhMG53 and hUC-MSCs could reduce the lesion volume and severity of neurologic deficit with the mNSS assessment, as well as improving the whole body condition [47]. Other neurological behavior assessments, including the MWM, FST, TST, SPT, NSF, and OFT, indicated that TBI mice exhibited motor and cognitive dysfunction, and also present some psychiatric-like anxiety and depression; however, these impairments were significantly alleviated after intravenous administration of rhMG53 and hUC-MSCs. Given the relatively short half-life of MG53, the protective effect of rhMG53 in TBI might be limited to delay the progression of TBI. However, rhMG53 can increase the survival of hUC-MSCs at the injury site, thereby improving the long-term therapeutic effect of hUC-MSCs to TBI.

Common secondary injuries associated with TBI induce elevated oxidative stress and cell apoptosis [48], which lead to impairment of cognition, motor function, and neurological behavior [49]. Previous studies indicated that human MSCs reduced ROS production and enhanced wound closure in a TBI in vitro model [50] and elicited therapeutic effects on TBI through the secretion of neurotrophic factors and the inhibition of apoptosis [51]. In addition, rhMG53 protects cardiomyocytes from H_2_O_2_-induced oxidative stress [15] and protects the heart from ischemic injury [52]. Furthermore, rhMG53 reduces ischemia/reperfusion injury to hepatocytes in vivo and in vitro [53]. Here, we showed that rhMG53 suppressed oxidative stress in cultured hUC-MSCs and TBI mice, accompanied by increased activity of SOD and decreased MDA content. We also found that rhMG53 and hUC-MSCs jointly rescued neural cells from apoptosis to alleviate brain injury.

GFAP is widely recognized as a specific biomarker for astrocytes which is now increasingly acknowledged as having fundamental and sophisticated roles in brain function and dysfunction by astrocyte-neuron interactions [54–57]. DCX is a microtubule-associated protein expressed in migrating and differentiating neurons, corresponding to the initial stage of neurogenesis [58]. NeuN is considered to be a reliable marker of mature neurons [59]. Our results showed that rhMG53 and hUC-MSCs boosted neurogenesis, with more GFAP^+^, DCX^+^, and NeuN^+^ cells presented in the hippocampus at different stages. Researchers have proposed that soluble factors secreted by MSCs facilitate the regenerative process of the injured neural tissue by enhancing proliferation, migration, and differentiation of native neural stem cells (NSCs) [60]. This was proved by the upregulated expression of neurotrophins (BDNF and NGF) in our study. However, the mechanism by which rhMG53 enhanced neurogenesis needs further exploration as there is limited knowledge about the way stem cells and rhMG53 target specific tissues.

PI3K/Akt-GSK3β signaling contributes to neuronal growth, proliferation, and differentiation of NSCs [61]; promotes hippocampal neurogenesis in a mouse model of Alzheimer’s disease [62]; and ameliorates the apoptosis of neuronal cells in a rat model of Parkinson’s disease [63]. ERK1/2 is a key regulator for neural differentiation [64], modulates neurogenesis [65] and neural apoptosis [66], and improves depression-like behaviors [67]. Activation of ERK and Akt pathways protected PC12 cells against oxidative stress [68], and MG53 activated PI3K/Akt-GSK3β and ERK1/2 signaling to elicit cardioprotective functions [15, 22]. In brain ischemia/reperfusion injury, rhMG53 increased the phosphorylation of Akt and GSK3β and exhibited neuroprotective function [20]. Exosomes secreted by MSCs could induce proliferation and migration of fibroblasts and H9C2 myocardial cells by activating Akt and ERK1/2 signaling cascades, which contributes to chronic wound healing and inflammation [69]. In the present study, we found that both rhMG53 and MSCs upregulated the phosphorylation of Akt, GSK3β, and ERK1/2 in the hippocampus of TBI mice, suggesting that activation of the pro-survival PI3K/Akt-GSK3β and ERK1/2 signaling might be one contributing factor for the beneficial effects of rhMG53 and hUC-MSCs for the TBI mice.

Although this study demonstrates that the co-treatment of rhMG53 and hUC-MCSs increases survival signaling in the TBI model, further studies are needed to assess the cellular mechanism that underlies reduced apoptosis, such as whether MG53 has an impact on the mitochondrial welfare of injured neurons under conditions of oxidative stress. Furthermore, hUC-MSCs secrete paracrine factors, which may be essential to understand how those paracrine factors may have a synergistic role with rhMG53 to mitigate TBI injury.

## Conclusions

Our findings show that rhMG53 can protect hUC-MSCs against oxidative damage, accompanied by enhanced proliferation and migration, and reduced apoptosis and senescence in vitro. In vivo, hUC-MSCs combined with rhMG53 were more effective in reducing the neurological deficits by protecting against neural oxidative stress, improving neurogenesis, and brain function in TBI mice by activating PI3K/Akt-GSK3β signaling. These findings provide a novel strategy of cell-based therapy, combining rhMG53 with hUC-MSCs for TBI treatment.

## Data Availability

The data that support the findings of this study are available from the corresponding author upon request.

## Abbreviations

BBB: Blood-brain barrier
DCX: Doublecortin
FST: Forced swim test
GFAP: Glial fibrillary acidic protein
hUC-MSCs: Human umbilical cord-derived mesenchymal stem cells
MDA: Malonyldialdehyde
mNSS: Modified neurologic severity score
MWM: Morris water maze
NeuN: Neuronal nuclei
NOR: Novel object recognition
NSF: Novelty suppressed feeding test
OFT: Open-field test
PBS: Phosphate-buffered saline
PI: Propidium iodide
qRT-PCR: Quantitative real-time PCR
rhMG53: Recombinant human MG53
ROS: Reactive oxygen species
SOD: Superoxide dismutase
SPT: Sucrose preference test
TBI: Traumatic brain injury
TRIM: Tripartite motif
TST: Tail suspension test
