# Correction to: The TRIM protein Mitsugumin 53 enhances survival and therapeutic efficacy of stem cells in murine traumatic brain injury

**DOI:** 10.1186/s13287-021-02598-x

**Published:** 2021-09-30

**Authors:** Fangxia Guan, Tuanjie Huang, Xinxin Wang, Qu Xing, Kristyn Gumpper, Peng Li, Jishi Song, Tao Tan, Greta Luyuan Yang, Xingxing Zang, Jiewen Zhang, Yuming Wang, Yunlei Yang, Yashi Liu, Yanting Zhang, Bo Yang, Jianjie Ma, Shanshan Ma

**Affiliations:** 1 School of Life Sciences, Zhengzhou University, Zhengzhou, 450001 Henan China; 2 The First Affiliated Hospital of Zhengzhou University, Zhengzhou, 450052 Henan China; 3 Henan Provincial People’s Hospital, No. 7 Weiwu Road, Zhengzhou, 450003 Henan China; 4 Department of Surgery, Davis Heart and Lung Research Institute, The Ohio State University, Columbus, OH 43210 USA; 5 Stuyvesant High School, 345 Chambers St, New York, NY 10282 USA; 6 Department of Microbiology and Immunology, Einstein College of Medicine, 1300 Morris Park Ave, Bronx, NY 10461 USA; 7 Department of Medicine and Neuroscience, Einstein College of Medicine, 1300 Morris Park Ave, Bronx, NY 10461 USA

## Correction to: Stem Cell Research & Therapy (2019) 10:352 10.1186/s13287-019-1433-4

The original article [1] contained errors in Figs. 2 and 5.

**Fig. 2:**
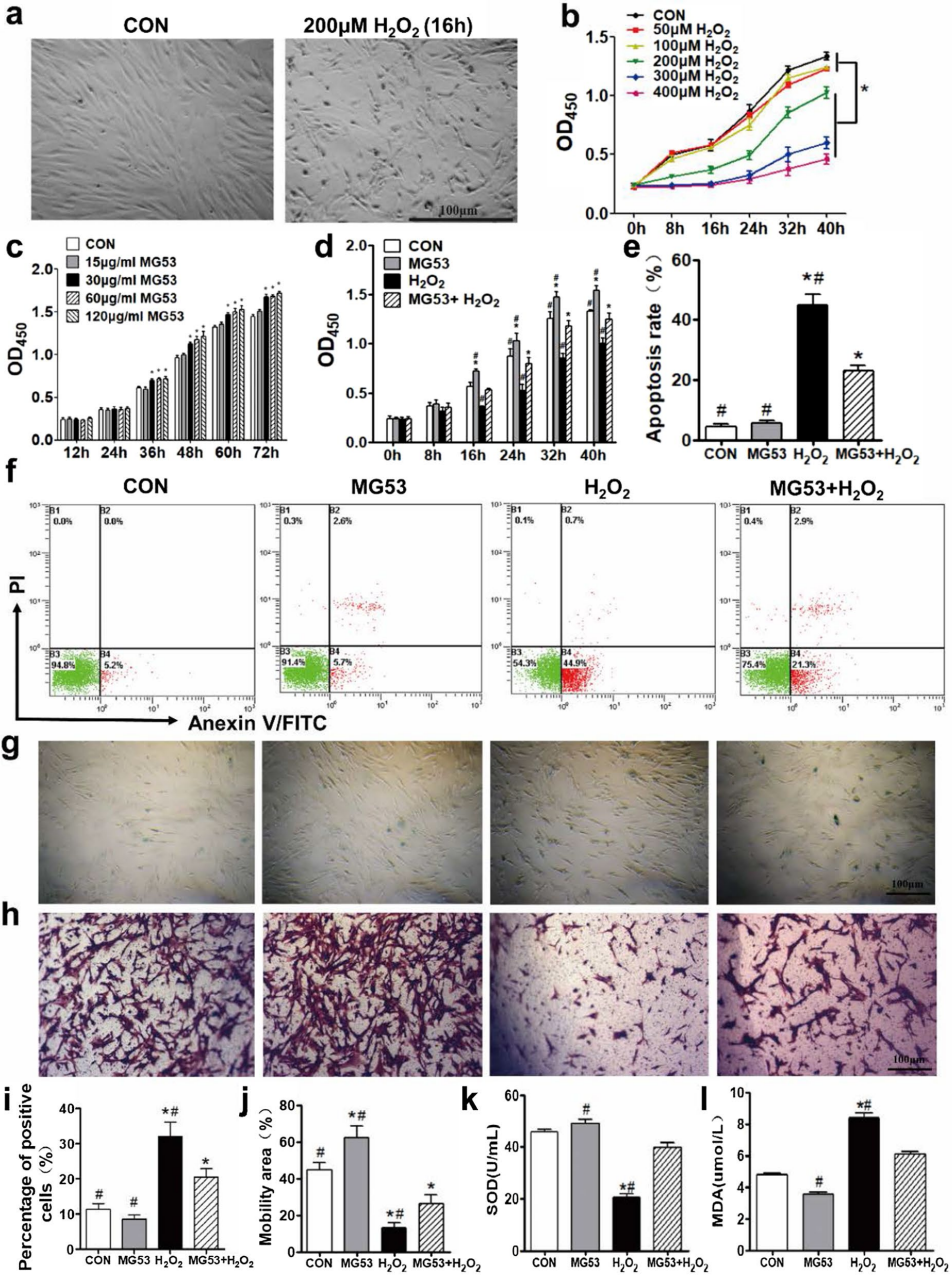
rhMG53 lessens H_2_O_2_-induced oxidative injury to hUC-MSCs and promotes cell migration. **a** Representative images of hUC-MSCs with and without 200 μM H_2_O_2_ treatment. **b** Time- and dose-dependent effects of H_2_O_2_ on hUC-MSCs. Cells were cultured in 0, 50, 100, 200, 300, or 400 μM H_2_O_2_, and OD450 was measured at 0, 8, 16, 24, 32, and 40 h post-treatment. Two hundred micromolar H_2_O_2_ was used for subsequent experiments to induce hUC-MSC oxidative damage. **c** Dose-dependent effects of MG53 on hUC-MSCs. Thirty micrograms per milliliter of rhMG53 was chosen for our in vitro experiments. **d** rhMG53 facilitates hUC-MSC proliferation and protects against H_2_O_2_-induced injury. **e** Quantification of apoptosis rate from Annexin V-FITC/PI flow cytometry. **f** Apoptosis of hUC-MSCs was detected and analyzed by Annexin V-FITC and PI double staining and flow cytometry as well. **g** Cell senescence was evaluated using a SA-β-gal kit. Senescent cells were dyed blue. **h** Transwell assay was used to assess cell migration. Migrated cells were stained with CV. Scale bar = 100 μm. Quantification of cell senescence (**i**) and migration (**j**). SOD activity (**k**) and MDA content (**l**) were measured from hUC-MSC lysates. Data are presented as mean ± SEM. n = 6 per group. *p < 0.05, compared with CON group; ^#^p < 0.05, compared with MG53 + H_2_O_2_ group

**Fig. 5:**
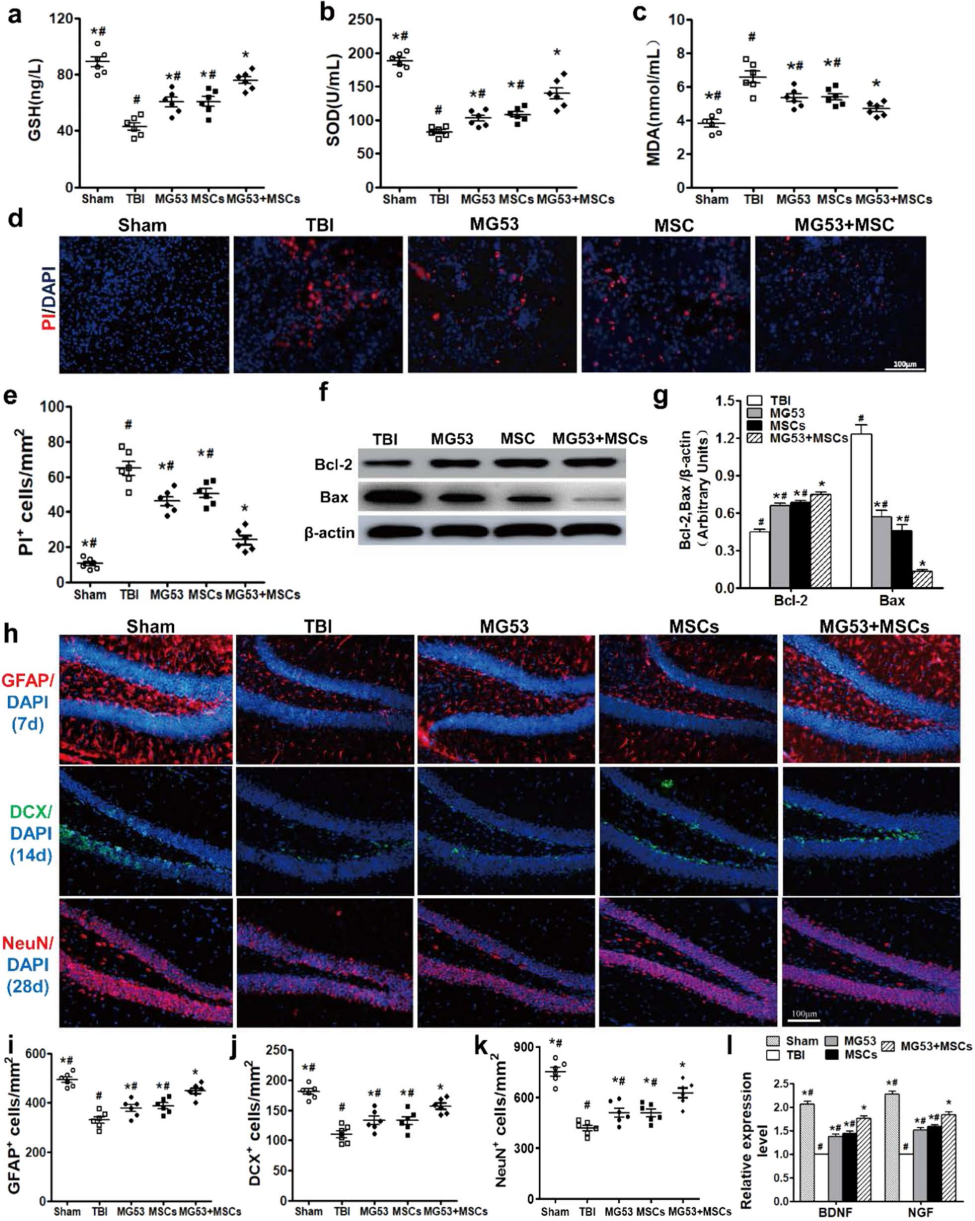
rhMG53 and hUC-MSCs reduce oxidative stress and cell death and increase neurogenesis after TBI. Quantification of the concentration of GSH (**a**) and SOD (**b**) and activity of MDA (**c**) at day 3 post-TBI. **d** PI staining in the cerebral cortex of TBI mice as a marker for cell death at 3 days post-TBI. Scale bar = 100 μm. **e** Quantification of the number of PI-positive cells in the four groups. Western blotting (**f**) and densitometric analysis (**g**) of Bcl-2 and Bax in the hippocampus of different TBI mice. **h** Immunofluorescence staining of GFAP + , DCX + , and NeuN + cells in the brain of the mice. Scale bar = 100 μm. Quantification of the number of GFAP + (**i**), DCX + (**j**), and NeuN + (**k**) cells in the four groups. **l** qRT-PCR for BDNF and NGF. Data were presented as mean ± SEM. n = 6 per group. *p < 0.05, compared with TBI group; ^#^*p* < 0.05, compared with MG53 + MSC group

In Fig. 2G, the representative SA-β-gal staining image of H_2_O_2_ group was mistakenly used for the MG53 + H_2_O_2_ group during assembly of the figure.

In Fig. 5H, the typical NeuN immunofluorescence staining image of MG53 was mistakenly used for the TBI group during assembly of the figure.

The authors have provided the correct figures and also reanalyzed the quantification of SA-β-gal staining (Fig. 2i) and NeuN immunofluorescence staining (Fig. 5 k).

The authors state that these mistakes do not affect the conclusion of the article.
